# The presence of lateral photophores correlates with increased speciation in deep-sea bioluminescent sharks

**DOI:** 10.1098/rsos.150219

**Published:** 2015-07-29

**Authors:** Julien M. Claes, Dan-Eric Nilsson, Jérôme Mallefet, Nicolas Straube

**Affiliations:** 1 Laboratoire de Biologie Marine, Earth and Life Institute, Université catholique de Louvain, Louvain-la-Neuve 1348, Belgium; 2 Lund Vision Group, Department of Biology, Lund University, Lund 22362, Sweden; 3 Bavarian State Collection of Zoology, Münchhausenstrasse 21, München 81247, Germany

**Keywords:** bioluminescence, diversification, evolution, shark, visual detection

## Abstract

The vast majority of species within the lanternshark genus *Etmopterus* harbour complex luminescent markings on their flanks, whose functional significance has long remained obscure. Recent studies, however, suggest these enigmatic photophore aggregations to play a role in intraspecific communication. Using visual modelling based on *in vivo* luminescence measurements from a common lanternshark species, we show that etmopterid flank markings can potentially work as a medium range signal for intraspecific detection/recognition. In addition, using molecular phylogenetic analyses, we demonstrate that the *Etmopterus* clade exhibits a greater than expected species richness given its age. This is not the case for other bioluminescent shark clades with no (or only few) species with flank markings. Our results therefore suggest that etmopterid flank markings may provide a way for reproductive isolation and hence may have facilitated speciation in the deep-sea.

## 1. Introduction

Sharks of the genus *Etmopterus* (Squaliformes: Etmopteridae) are among the most intriguing bioluminescent organisms. Their tiny photogenic organs contain an unknown light-producing chemistry [1], are controlled by a unique ‘hybrid’ physiological control involving both hormonal and neural components [2], and form a diversity of patterns whose function has puzzled ichthyologists for more than a century [3–5]. Recent advances, however, suggest their luminescence to be a versatile tool involved in varied behaviours including camouflage by counterillumination [6,7], aposematism [8] and intraspecific signalling [9–11]. While counterillumination and aposematism are well supported by behavioural, experimental and theoretical data, intraspecific signalling is currently only corroborated by circumstantial evidences i.e. sex- or clade/species-specific difference in bioluminescent patterns, especially at the level of the lateral photophore areas (‘flank markings’). Although they can be grouped into five main patterns, these bioluminescent markings are highly variable across species [12,13]. As a consequence, scientists have suggested these areas to favour sympatric speciation by promoting reproductive isolation [7,11]. Interestingly, species-specific bioluminescence has been recently demonstrated to increase speciation rate in deep-sea bony fishes [14]. A similar situation could explain the evolutionary success of the genus *Etmopterus*, which, with 38 currently described species, is among the most prolific shark genera [15].

In this work, we first use a recent theory for pelagic vision to test whether etmopterid bioluminescent flank markings could efficiently work as an intraspecific recognition device. Subsequently, we performed molecular phylogenetic analyses to test whether the presence of these lateral photophore areas correlates with a higher diversification rate within bioluminescent sharks as was demonstrated for myctophids, which also show lateral photophores [16].

## 2. Material and methods

### 2.1 *In vivo* bioluminescence recordings

Specimens from a common etmopterid species (*Etmopterus spinax*) were collected in the Raunefjord and transferred to seawater tanks placed in a dark cold (4°C) room at Espeland Marine Station (Norway).

A luminometer (Berthold FB12, Pforzheim, Germany) coupled to an optical fibre allowed *in vivo* recording of ventral and lateral luminescence intensities from several live specimens according to Claes *et al*. [6]. Values were corrected for fibre absorption and angular losses. For modelling purpose, all photophores from a single shark specimen were considered to have exactly the same intensity. This intensity was calculated by dividing lateral light output by lateral photophore density, which was estimated under a binocular microscope according to Claes *et al*. [10]. Photophore spacing was determined from photophore density assuming a square mosaic.

### 2.2 Visual modelling

The detection distances of lateral glows were calculated according to the theory developed by Nilsson *et al*. [17]. This distance depends on the intensity of downwelling daylight, thus on water depth and observer sighting direction. Therefore, as in Claes *et al*. [8], *E. spinax* was assumed to occur at ‘counterillumination depth’ where its silhouette, cloaked by ventral photophores, is invisible from below [6], and sighting direction was chosen to be horizontal given the body position of etmopterid lateral luminescent markings. Counterillumination depth was determined using the mean spacing (0.248 mm) and mean intensity (2.52×10^6^ photons s^−1^) of ventral photophores from shark ‘*α*’ (a 43 cm total length (TL) male specimen that exhibited the brightest flank markings of our dataset) as inputs in the eqn 7 in Supplemental Information from Nilsson *et al*. [17]. Beam attenuation and back-scattering coefficients were set to 0.3 m^−1^ and 0.0385 m^−1^, respectively, to agree with the turbid waters of the fjords according to Nilsson *et al*. [16]. The horizontal detection distance of specimen *α* flank markings (photophore mean spacing=0.191 mm; photophore mean intensity=2.52×10^6^ photons s^−1^), modelled as an extended bioluminescent source seen against a transparent background (the downwelling daylight), was subsequently calculated for a series of pupil diameters (0–150 mm) according to Nilsson *et al*. [17]. Measurements performed in a complete ontogenetic series further provided a precise delimitation of the shark's pupil diameter range. Photoreceptor cell diameter was set to 3 μm according to recent measurements of *E. spinax* rod photoreceptor [18].

### 2.3 Species richness curve

For estimating diversification and relative extinction rates, we used MEDUSA [19] implemented in the R module GEIGER [20]. Rate estimates are based on the chronogram in Straube *et al*. [11] pruned to squaliform sharks. The background diversification and extinction rates estimated in MEDUSA were subsequently used to calculate crown and stem limits during the last 70 Ma (5 Ma increments) using the bd.ms module in GEIGER [21]. The resulting data allowed generation of an expected species richness curve from different points in time. Using the crown.p and stem.p options of the bd.ms module in GEIGER [20,21], we further calculated the probabilities of obtaining the current bioluminescent shark clades *Etmopterus*, *Centroscyllium* and *Aculeola*, *Trigonognathus*, and Dalatiidae given species number, age, diversification rate *r* and extinction rate *ε*. Species numbers were derived from Pollerspöck & Straube [15] and clade ages refer to Straube *et al*. [11]; see the electronic supplementary material for details on the performed analysis.

## 3. Results

**Figure 1.**
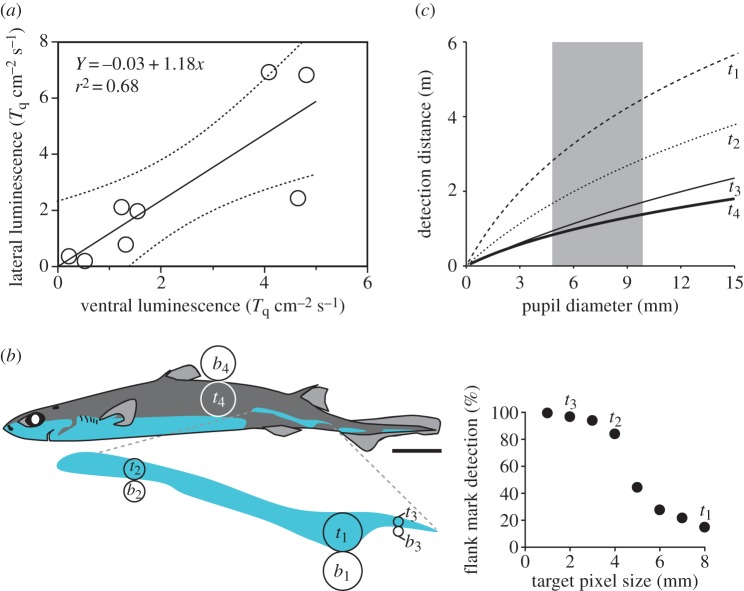
*In vivo* recordings and visual modelling of *Etmopterus spinax* luminescence. (*a*) Correlation between lateral and ventral (counterilluminating) photophore luminescence intensity. (*b*) Target (*t*) and background (*b*) pixels used in the theoretical visual modelling: *t*_1_=bioluminescent detection pixel, *t*_2_=gross discrimination pixel (allowing more than 50% of the lateral pattern to be seen), *t*_3_=fine discrimination pixel (allowing more than 95% of the lateral pattern to be seen), *t*_4_=non-bioluminescent (black) detection pixel. Scale bar, 5 cm. (*c*) Detection distance of target pixels at counterillumination depth according to the observer's pupil diameter. The shaded area represents the pupil diameter range observed from a complete *E. spinax* ontogenetic series.

Luminescence from ventral and lateral (flank markings) photophores was recorded in eight adult *E. spinax* specimens (35.5–49.5 cm TL). Light intensities from these photogenic structures, which are under the same hormonal control, were significantly correlated (*p*=0.0116; figure 1*a*). Ventral photophore intensity, combined with the visual theory for pelagic vision developed by Nilsson *et al*. [16,17], allowed us to determine the counterillumination depth of specimen *α*, i.e. 203 and 291 m for heavily overcast and clear skies (sun at 45°), respectively. Using Nilsson *et al*.'s theory again but with lateral photophore intensity and pupil diameter measurements from a complete ontogenetic series of *E. spinax* (*n*=40; 12–55 cm TL), we then determined that, at counterillumination depth, flank markings of specimen *α* are detectable by conspecifics at 2.8–4.4 m, well resolved at 1.7–2.9 m and fully resolved at 1–1.7 m (figure 1*b*,*c*). In the absence of lateral photophores, specimen *α* would only be detected by conspecifics at 0.9–1.4 m (figure 1*b*,*c*).

**Figure 2.**
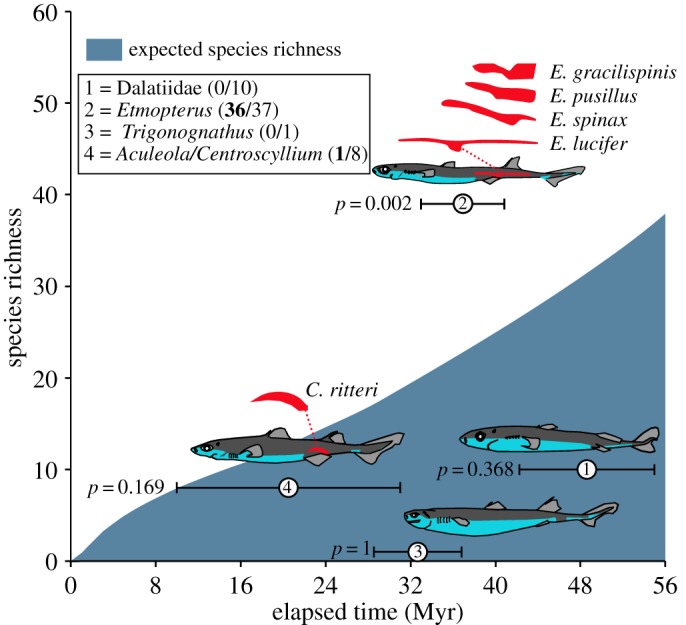
Expected species richness curve of bioluminescent sharks indicating the 95% CI for the expected number of species given clade age. Values in parentheses indicate, for each clade, the ratio between the number of species with flank markings (bold) versus the total species number. Only the *Etmopterus* clade, which contains the highest proportion of species with flank markings (97%), shows a significantly (*p*<0.01) higher species richness than expected. Flank markings are represented in red on shark drawings and on insets above them (enlarged view). To illustrate the morphological diversity of flank markings within *Etmopterus* genus, the flank marking shapes of four species were represented.

MEDUSA estimated a background diversification rate *r* of 0.017 and a relative extinction rate *ε* of 0.82. A rate shift was detected at the split of the genus *Etmopterus*, increasing the net diversification rate *r* to 1. The *Etmopterus* clade exhibits exceptional species diversity given its age, whereas all other clades, which contain no or a minority of species with flank markings, fall within the 95% CI of expected species diversity given their ages (figure 2).

## 4. Discussion

Contrary to the photophores of other luminescent animals, which are often sparsely distributed or isolated, shark photophores form extended photogenic areas. Because they produce homogeneous bioluminescent emissions, these areas, when they occupy a ventral position, are particularly efficient to camouflage a silhouette [6,7,22]. Even though *Etmopterus* species display such ventral counterilluminating areas, they also display extended bioluminescent areas on their lateral sides, the so-called flank markings, which are hypothesized to be involved in intraspecific signalling [9–11]; their lateral body position and light kinetics (glow) indeed prevent them from being used in other bioluminescent behaviours such as camouflage, defence or prey capture. Collecting behavioural data demonstrating bioluminescent communication in elusive deep-sea animals such as bioluminescent sharks is logistically challenging. However, every luminous signal requires a target photoreceptor to be ecologically relevant [23]. This postulate allowed us to investigate bioluminescent shark communication via a physical modelling based on a recent theory for pelagic vision [18]. Using *E. spinax* as a model lanternshark species, this work demonstrates for the first time that these areas can be finely resolved at relatively long distance by conspecifics (figure 1*c*). Etmopterid flank markings exhibit a remarkable diversity of shapes that appears to be genetically supported [11]. When glowing, possibly in association with other clues (e.g. other bioluminescent areas), these lateral structures can therefore impact species recognition and potentially sexual selection in the darkness of the deep sea, as previously suggested [9,11]. Our theoretical approach also reveals that these markings are highly visible to predators with large pupils (e.g. large piscivorous fishes and marine mammals). Interestingly, captive *E. spinax* specimens often rotate their body right and left while swimming. This behaviour allows the continuous but directional photophore luminescence to generate intermittent signals similar to those of communicating fireflies or flashlight fishes, which are less conspicuous to predators [24,25].

From an evolutionary point of view, etmopterid flank photophores are considered to be an exaptation of ventral counterilluminating photophores [8]. In that context, the migration of ventral photophores towards the flanks would have occurred in the Palaeogene, potentially as an adaptation for bioluminescent signalling, probably during a deep-sea colonization event [7,11]. Here, we show that the extant *Etmopterus* clade exhibits a greater species richness than expected given its age, which is not the case for other bioluminescent shark clades with no (or only few) species with flank markings. Although the rapid diversification of *Etmopterus* sharks might be linked to a combination of different factors, our results agree with the idea that etmopterid flank markings are analogous to lateral photophores of lanternfishes (myctophids) [14], which may provide a way for reproductive isolation and facilitation of speciation in the darkness of the deep open oceans.

## Supplementary Material

Supplementary text: detailed materials and methods for phylogenetic analyses. Supplementary Fig. 1: Plotted results from the MEDUSA analysis with species level representation. Supplementary Table 1: Species richness values (from [15]) for the different shark clades included in the analyses. Supplementary Table 2: Supplementary Table S2. Crown limits calculated using a background diversification rate r = 0.017 and an extinction rate e = 0.82. This allows to draw the axpected species richness curve of bioluminescent sharks present in Figure 2 of the main paper. Supplementary Table S3: Input information for calculating the probability of attaining the four clades Etmopterus, Centroscyllium & Aculeola, Dalatiidae and Trigonognathus. Supplementary Table S4. In vivo luminescence raw data. Supplementary Reference: one supplementary references cited in the electronic supplementary material but not in the main paper.

## Data accessibility

All data supporting the paper are presented either in the main manuscript or in the electronic supplementary material.
