# Supplementary material for: The presence of lateral photophores correlates with increased speciation in deep-sea bioluminescent sharks
Source: R Soc Open Sci. 2015 Jul 29;2(7):150219. doi: 10.1098/rsos.150219 (PMC4632593; doi:10.1098/rsos.150219)
Supplement: Supplementary text: detailed materials and methods for phylogenetic analyses. Supplementary Fig. 1: Plotted results from the MEDUSA analysis with species level representation. Supplementary Table 1: Species richness values (from [15]) for the different shark clades included in the analyses. Suppleme [file rsos150219supp1.docx]

**Electronic supplementary material**

**The presence of lateral photophores correlates with increased speciation in deep-sea bioluminescent sharks**

Julien M. Claes, Dan-Eric Nilsson, Jérôme Mallefet and Nicolas Straube

**Text, additional figure (S1), tables (S1-S3) and reference (S1)**

**Detailed Materials and Methods for Phylogenetic Analyses**

**1. Estimation of background diversification rate, significant rate shifts and extinction rate.**

The software package MEDUSA implemented in the R package GEIGER was used to estimate diversification and extinction rates as well as significant changes in the diversification rates. Please see [19−21] as well as the Geiger manual for details on the different options and examples on its usage. Provided input for our analysis were the chronogram from [11]. The timetree originally has 74 tips including outgroups. In a first step, we pruned outgroups from the tree (*Chimaera, Odontaspis, Apristurus*) in R so that the chronogram contained Squaliform sharks only. Thereafter, we updated the taxon names, so that tree tips represent only a single species in the tree resulting in a tree with 43 tips. This step included re-evaluating the taxonomic status of some taxa names (see Supplementary Table S1) as well as tree-pruning, if multiple tips represented a single species.

The second input refers to species richness values, which need to be provided, if the input tree is not completely sampled. Species richness values for the different Squaliform lineages were attained from the online database shark-references.com [15]. Species richness values refer to the described, valid species in different squaliform families. Species missing in the tree were assigned in the species richness values table to the representative lineages in the timetree attained from [11]. See Supplementary Table S1 for species richness values and Supplementary Fig. S1 for an overview of the results derived from the MEDUSA analysis.

**2. Species richness curve**

The resulting background diversification and extinction rates estimated with MEDUSA were subsequently used to calculate crown and stem limits every 5 Ma for a total of 70 Ma using the bd.ms module [20, 21] implemented in the GEIGER toolkit. See Supplementary Table S2 for calculated data-points underlying the species richness curves.

**3. Probabilities of extant luminescent taxa.**

We used the crown p options of the bd.sm module implemented in the GEIGER package to calculate the probabilities to obtain the clades *Etmopterus*, *Trigonognathus*, *Centroscyllium* & *Aculeola*, and Dalatiidae representing extant luminescent taxa. Input information comprises species richness (see Supplementary Table S3), clade age deferred from [11], diversification rate *r* and extinction rate *ε*.

**Supplementary Fig. S1**. Plotted results from the MEDUSA analysis with species level representation. Background diversification rate *r* = 0.017, extinction rate *ε* = 0.8. Significant rate shift indicated by red lines at diversification of *Etmopterus* (*r* = 0.1). Aicc chosen as stopping criterion. Appropriate aicc-threshold = 2.4.

**Supplementary Table S1**. Species richness values (from [15]).

| lineage represented in tree | assigned species number | remark |
| --- | --- | --- |
| *Centrophorus squamosus* | 12 | This lineage is assumed to represent the complete *Centrophorus* lineage comprising 12 species, i.e. *C. squamosus* (sampled in the tree)*, C. artromarginatus, C. granulosus, C. harrisoni, C. isodon, C. lucitanicus, C. moluccensis, C. seychellorum, C. tesselatus, C. uyato, C. westraliensis* and *C. zeehani.* |
| *Deania calcea* | 4 | *D. calcea* represents the *Deania lineage which currently contains 4 species,* i.e. *D. calcea, D. profundorum, D. quadrispinosa* and *D. hystricosa.* |
| *Dalatias licha* | 4 | In total, nine *Dalatiidae* species are described; species richness value codes for *Dalatias* and also refer to *Isistius* specie*s (I. brasiliensis* and *I. plutodes)* and *Mollisquama parini,* as a sister-group relationship of *Dalatias, Isitius* and *Mollisquama* was phylogenetically estimated recently (1)*.* |
| *Squaliolus aliae* | 5 | *Squaliolus* represents a lineage here, which is further assumed to include 4 other species, i.e*. S. laticaudus, Euprotomicrus bispinatus, Heteroscymnoides marleyi* and *Euprotomicroides zentedeschia.* |
| *Scymnodon plunketi* | 1 |  |
| *Scymnodon ringens* | 3 | This lineage is also assumed to include species *S. ichiharai* and *S. obscurus.* |
| *Centroscymnus owstoni* | 4 | *C. owstoni* represents the *Centroscymnodon* lineage here which currently contains 4 species *(C. owstoni, C. macracanthus, C. cryptacanthus* and *C. coelolepis).* |
| *Centroselachus crepidater* | 2 | This lineage is assumed to represent the *Centroselachus* as well as *Zameus* lineage. |
| *Oxynotus paradoxus* | 5 | There are five species of *Oxynotus* currently described*: O. bruniensis, O. paradoxus, O. japonicus, O. caribbaeus* and *O. centrina.* |
| *Centroscyllium fabricii* | 2 | This lineage is assumed to also represent the other Atlantic *Centroscyllium* species known*, C. excelsum.* |
| *Centroscyllium ritteri* | 2 | This lineage is assumed to also represent the other Pacific *Centroscyllium* species known*, C. kamoharai.* |
| *Centroscyllium granulatum* | 1 |  |
| *Centroscyllium nigrum* | 2 | This lineage is assumed to also represent *C. ornatum.* |
| *Aculeola nigra* | 1 |  |
| *Etmopterus bigelowi* | 1 |  |
| *Etmopterus pusillus* | 5 | This lineage is further assumed to represent *E. schmidti, E. splendidus, E. carteri* and *E. caudistigmus* as non-sampled taxa from the *E. pusillus* clade. |
| *Etmopterus joungi* | 1 |  |
| *Etmopterus fusus* | 1 |  |
| *Etmopterus pseudosqualiolus* | 1 |  |
| *Etmopterus sentosus* | 1 |  |
| *Etmopterus sculptus* | 1 |  |
| *Etmopterus cf. lucifer* | 1 |  |
| *Etmopterus lucifer* | 5 | This lineage is further assumed to represent *E. bullisi E. pycnolepis, E. evansi and E. burgessi,* as non-sampled taxa from the *E. lucifer* clade. |
| *Etmopterus dislineatus* | 1 |  |
| *Etmopterus brachyurus* | 1 |  |
| *Etmopterus molleri* | 1 |  |
| *Etmopterus sheikoi* | 1 |  |
| *Etmopterus virens* | 1 |  |
| *Etmopterus polli* | 1 |  |
| *Etmopterus schultzi* | 1 |  |
| *Etmopterus gracilispinis* | 3 | This lineage is further assumed to represent *E. robinsi* and *E. perryi as* non-sampled taxa from the *E. pusillus* clade. |
| *Etmopterus* sp. B | 1 |  |
| *Etmopterus viator* | 1 |  |
| *Etmopterus spinax* | 3 | This lineage is further assumed to represent *E. tasmaniensis, E. litvinovi* and *E. hillianus* as non-sampled taxa from the *E. spinax* clade. |
| *Etmopterus princeps* | 1 |  |
| *Etmopterus compagnoi* | 1 |  |
| *Etmopterus granulosus* | 1 |  |
| *Etmopterus unicolor* | 1 |  |
| *Etmopterus dianthus* | 1 |  |
| *Trigonognathus kabeyai* | 1 |  |
| *Somniosus microcephalus* | 4 | This lineage is further assumed to represent *S. rostratus, S. pacificus,* and *S. antarcticus.* |
| *Squalus megalops* | 28 | This lineage is further assumed to represent *S. acanthias, S. albifrons, S. altipinnis, S. blainville, S. brevirostris, S. bucephalus, S. chlorocullus, S. crassispinus, S. cubensis, S. edmundsi, S. formosus, S. grahami, S. graffini, S. hemipinnis, S. japonicus, S. lalannei, S. melanusrus, S. mitsukurii, S.montalbani, S. nasutus, S. notocaudatus, S. rancureli, S. raoulensis* and *S. suckleyi.* |

**Supplementary Table S2**. Crown limits calculated using a background diversification rate *r* = 0.017 and an extinction rate *ε* = 0.82.

| Mio a | lower bound | upper bound crown |
| --- | --- | --- |
| 1 | 0.9964 | 0.8941 |
| 5 | 1.2778 | 6.0320 |
| 10 | 1.0890 | 8.3937 |
| 15 | 1.0894 | 10.8082 |
| 20 | 1.1017 | 13.3720 |
| 25 | 1.1181 | 16.1275 |
| 30 | 1.1371 | 19.1038 |
| 35 | 1.1585 | 22.3265 |
| 40 | 1.1820 | 25.8203 |
| 45 | 1.2079 | 29.6107 |
| 50 | 1.2361 | 33.7245 |
| 55 | 1.2669 | 38.1907 |
| 60 | 1.3004 | 43.0402 |
| 65 | 1.3370 | 48.3066 |
| 70 | 1.3767 | 54.0261 |

**Supplementary Table S3**. Input information for calculating the probability of attaining the four clades *Etmopterus*, *Centroscyllium & Aculeola*, Dalatiidae and *Trigonognathus*.

|  | *Etmopterus* | *Centroscyllium/ Aculeola* | Dalatiidae | *Trigonognathus* |
| --- | --- | --- | --- | --- |
| age | 36.48 | 22.7 | 44.83 | 40.6 |
| interval | 31.55–41.36 | 12.48–38.78 | 52.79–68.71 | 35.70–46.02 |
| nr species | 38 | 8 | 9 | 1 |
| *r* | 0.0165548 | 0.0165548 | 0.0165548 | 0.0165548 |
| *ε* | 0.821943 | 0.821943 | 0.821943 | 0.821943 |
| crown p | 0.00189 | 0.16947 | 0.36806 | 1 |

**Supplementary Reference**

1. Grace, MA, Doosey, MH, Bart, HL, Naylor, GJ. 2015 First record of *Mollisquama sp.*(Chondrichthyes: Squaliformes: Dalatiidae) from the Gulf of Mexico, with a morphological comparison to the holotype description of *Mollisquama parini* Dolganov. *Zootaxa*, **3948**, 587-600. (doi:[10.11646/zootaxa.3948.3.10](http://dx.doi.org/10.11646/zootaxa.3948.3.10))
